# Detection of *Borrelia burgdorferi* Sensu-Lato-Specific Antibodies in Sera of Canine and Equine Origin—A Comparative Study with Two Line Immunoassays

**DOI:** 10.3390/vetsci9110633

**Published:** 2022-11-14

**Authors:** Sophie Charlotte Doff, Jasmin Wenderlein, Anna Wiesinger, Stephanie Hiereth, Sebastian Ulrich, Reinhard K. Straubinger

**Affiliations:** Chair for Bacteriology and Mycology, Institute for Infectious Diseases and Zoonoses, Department of Veterinary Sciences, Faculty of Veterinary Medicine, LMU Munich, Veterinärstr. 13, 80539 Munich, Germany

**Keywords:** antibody, *Borrelia burgdorferi* sensu lato, canine, equine, serum diagnosis, line immunoassay

## Abstract

**Simple Summary:**

The causative agents of Lyme borreliosis, spirochetes from the *Borrelia burgdorferi* sensu lato complex, can be transmitted via various life stages of the tick, making this disease a serious concern pertaining to One Health approaches. The detection of specific antibodies against *Bb*sl is generally achieved by using a two-tiered test approach based on an ELISA combined with a line immunoassay (LIA). In this study, canine and equine serum samples with known antibody status were tested with two different LIAs. Results were compared in term of sensitivity, specificity, diagnostic outcome for dogs and horses, as well as of operability of the test. For canine serum samples, reliable results can be achieved with both LIAs. In contrast, the serodiagnosis of horses is still challenging, and improvements of both LIAs are recommended.

**Abstract:**

Lyme borreliosis is a vector-borne disease in humans and animals caused by bacteria from the *Borrelia burgdorferi* sensu lato complex (*Bb*sl). The possible transmission of *Bb*sl from companion animals to humans via ticks makes this disease important in terms of One Health approaches. Thus, early and accurate diagnosis and treatment are of utmost importance. Today’s standard for the detection of specific antibodies against *Bb*sl is a two-tiered test system based on an ELISA for screening combined with a line immunoassay (LIA) for confirmation. In this study, 200 canine and 200 equine serum samples with known antibody status were tested with two different LIAs (A and B). Results were compared regarding sensitivity, specificity, the diagnostic outcome for dogs and horses, as well as operability of the test. The results for canine serum samples corresponded to 94.0%, making both LIAs a good choice for LB diagnostic in dogs. For equine serum samples, the agreement of both tests was 65.5%, displaying the challenge equine samples still provide in LB diagnostic. Major concerns were the interpretation of the OspA antigen (AG) signal and the use of unspecific (i.e., p100/p83) or too sensitive signals on the LIA. The operability of both LIAs was equally user-friendly. Regarding the tests’ evaluation, the scanning process provided by LIA A was a major advantage considering the comparability of the tests.

## 1. Introduction

Lyme borreliosis (LB) is the most diagnosed human vector-borne disease in Europe and Northern America. The infection occurs in dogs and horses and is caused by spirochetes from the *Borrelia burgdorferi* sensu lato complex (*Bb*sl). Seroprevalence for *Bb*sl-specific antibodies in dogs varies between 0.1% and 15.0% worldwide [1,2,3], while in horses, it ranges from 8.0% to 33.0% [4,5].

There are several diagnostic methods for detecting borrelial infections in mammalian hosts. Culture of *Borrelia* spp. is complicated and may take up to six weeks [6,7,8]. The detection of *Bb*sl-specific DNA via polymerase chain reaction (PCR) is discussed controversially, as low numbers of borreliae in tissues of infected hosts might lead to false-negative results [9,10,11]. Since the direct detection of borrelial organisms is difficult and prone to erratic results [12], indirect tests detecting *Bb*sl-specific antibodies are the method of choice in LB diagnostics [13]. The first serologic test used was the indirect immunofluorescence antibody test (IFAT), which contained a broad spectrum of antigens [12,14,15]. Due to low specificity [16] and the inability to discriminate between infected and vaccinated individuals [17], further test systems such as ELISAs [16,18] and Western blots [19] have been developed [20]. Western blots (WB) were established to detect well-characterized antibodies, which are highly specific for the serologic status [19]. Proteins are blotted on nitrocellulose membranes and bind *Bb*sl-specific antibodies of the patient’s serum. Those immune complexes are subsequently visualized by color reactions on the carrier [19]. This diagnostic process allows the specific detection of borrelial infections and the differentiation between vaccinated and infected patients [21,22]. Suitable antigens for immunoblotting are the following proteins (p): p18/p21 (decorine binding protein A, DbpA), p23/24 (outer surface protein C, OspC), p28, p30 (OspA), p39 (borrelia membrane protein A, BmpA), p41, p43, p45, p58, p66, p83, p93, and p100 [13,23,24]. Commonly, these proteins are derived from *B. afzelii* and show a broad cross-reactivity with antibodies induced also by other species of the *Bb*sl-complex [25,26]. The detection of the lipoprotein variable major protein-like sequence expressed (VlsE) in serologic diagnostic procedures contributes to an even higher specificity of these tests [27]. The VlsE gene contains a lipoprotein leader sequence, a N- and C-terminal unique conserved region, and a *vls* cassette with variable (V1–V6) and invariable regions (IR1–IR6) [28,29]. The expression of the VlsE lipoprotein occurs only in vivo by metabolically active *Bb*sl organisms and is a highly specific marker for infection [27].

The current diagnostic standard for LB is based on a two-tiered test system in which an ELISA with a high sensitivity serves as the first screening step [30,31]. A kinetic ELISA (KELA) is a diagnostic method in ELISA format, and its usability and the possibility of automatization contribute to its popularity [30,32]. Subsequent verification of KELA-positive samples and the differentiation between vaccination and infection status is achieved by immunoblotting the samples using assays such as the line immunoassay (LIA). LIAs were developed to standardize reactions and simplify the diagnostic process. Hence, recombinant antigens are sprayed on membranes as single antigen (AG) lines. The specificity of these semi-quantitative line immunoblots is very high, allowing differentiation between vaccinated and naturally infected animals [33].

In this study, we compared two LIAs by testing them with canine and equine serum samples obtained from animals with known antibody status against *Bb*sl. A regular reconsideration of available test systems is detrimental, as science advances and new insights and opportunities should be met with the diagnostic methods available. As the treatment of LB is not trivial and the silent infection of canines and equines with *Bb*sl-complex species is a One Health concern, the appropriateness of diagnostic methods used to detect antigens against *Bb*sl-complex species should be verified regularly. Results were compared in terms of sensitivity, specificity, and diagnostic outcome to emphasize the benefits and detriments of each LIA. Further, the handling, operability, and evaluation procedures were evaluated.

## 2. Materials and Methods

### 2.1. Serum Samples

A total of 400 serum samples were assessed for *Bb*sl-specific antibodies with an in-house KELA and two LIAs. The collection of sera consisted of 200 canine and 200 equine samples (Table 1). The sera were sent to the Chair for Bacteriology and Mycology of the LMU Munich for diagnostic purposes or were available from previous experiments and thereafter stored at −80 °C. All tested sera were from animals showing clinical signs matching those of LB, and antibiotic treatment of animals needed precursory serologic confirmation. Those serum samples were tested with the in-house KELA and the Borrelia Veterinär plus OspA LINE (VIROTECH Diagnostics GmbH, Ruesselsheim, Germany) or its predecessor model for past research or diagnostic purposes. For the conduction of this study, residuals from these canine and equine serum samples with matching antibody status determined in the previous diagnostic testing (i.e., seropositive, seronegative, equivocal, or vaccinated) were selected. However, the testing was based on antibody detection only, and the presence of *Borrelia* spp. organisms was not determined. To avoid biases due to possible false allocation in the previous serologic testing, two control groups were included in this study. The “canine control sera” consisted of serum samples from dogs with a confirmed *Bb*ss infection status (i.e., “positive”), vaccinated (i.e., “vaccination”), or specific-pathogen free (i.e., “negative”) [34,35,36]. The “equine control sera” originated from a vaccination study with horses using the vaccine EquiLyme^®^ (Boehringer Ingelheim Vetmedica GmbH, Ingelheim am Rhein, Germany) [37].

All serum samples described in this study were again tested for quantitative antibody levels with the in-house KELA and afterward analyzed using the two LIAs further named LIA A and LIA B. For the serologic testing, the sera were thawed and stored at +8 °C for a maximum of six hours between the tests.

### 2.2. Antibody Detection in the Serum Samples

The KELA used in this study to measure “total *Bb*sl-specific” and “OspA-specific” antibody levels was conducted as published previously [31,32].

#### 2.2.1. LIA A

LIA A is a line immunoassay for semi-quantitative detection of specific antibodies against *Bb*sl-complex species. The detection of antibodies was performed according to the manufacturer’s instructions with the supplied nitrocellulose strips. All antigens present on the strips are listed in Table 2 along with the genospecies from which they were derived.

All chemical components needed for the antibody detection with LIA A were brought to room temperature before use. First, the wash and incubation buffer (WIB) were prepared by mixing the buffer concentrate with aqua destillata (dH_2_O) in a ratio of 1:5. Then, 1.5 mL of the WIB was added to each well of a ten-welled incubation tray. One test strip for each tested serum was then placed in one of these incubation wells. The incubation tray was placed on the rocking shaker for five minutes until all strips were fully moistened. Afterward, 15 µL of the serum sample was added to each well and incubated for 45 min on the rocking shaker. After carefully draining all fluids, 1.5 mL of WIB was added per well. After five minutes of incubation on the rocking shaker, all fluids were poured off; this washing step was repeated three times in total. Then, 1.5 mL of the conjugate was added to each well and incubated on the rocking shaker for 45 min followed by three washing steps as described above. After draining all liquids, 1.5 mL of the substrate was added to each well. After an incubation for ten minutes followed on the rocking shaker, all fluids were removed, and the strips were washed three times with 1.5 mL of dH_2_O. The strips were then carefully removed from the wells and placed on a clean, absorptive paper to dry for at least 20 min.

The LIA strips were evaluated using the manufacturer’s scanner and software version 5.1.2. with values from 0 to 0.9 (negative), 1.0 (equal to cut-off control (COC)), and greater than 1.0 (positive). Values have then been automatically assigned to a result by the scanner (Table 3).

#### 2.2.2. LIA B

LIA B was developed for semi-quantitative detection of *Bb*sl-specific antibodies. All antigens present on the strips are listed in Table 2 along with the genospecies from which they were derived. Antibody detection with the supplied LIA strips was performed according to the manufacturer’s instructions. All components were brought to room temperature before use. The washing buffer concentrate was mixed 1:10 with dH_2_O. For each tested serum sample, one supplied strip was placed in an incubation well that is part of a supplied incubation tray consisting of eight wells. Compounded washing buffer (1.5 mL) was added to each reaction well; the incubation tray was then placed on the rocking shaker to thoroughly moisten the LIA strips for one minute. Next, 15 µL of serum was added to each well and incubated for 30 min on the rocking shaker. After incubation, fluids were carefully poured off. The strips were washed with 1.5 mL of washing buffer for five minutes and three times in a row while placed on the rocking shaker. For the next step, the IgG-conjugate was diluted at 1:100 with the washing buffer, and 1.5 mL of the compounded conjugate mix was dispensed into each well and incubated for 30 min on the rocking shaker. After pouring off all fluids, washing steps were repeated three times as described above and all fluids were drained carefully. Subsequently, 1.5 mL of dH_2_O was added to each well, incubated for one minute, and then removed. Then, 1.5 mL of the substrate was added to each well and incubated for twelve minutes. After removing the substrate from the incubation wells, the substrate reaction was stopped by adding 1.5 mL of dH_2_O to the incubation well for one minute, and thereafter, all fluids were poured off. This step was repeated three times. All fluids were then drained, and the strips were carefully removed from the incubation wells and transferred to a clean, absorptive paper for drying.

Evaluation of the strips was carried out visually by the examiner; reactions were categorized according to the AG signal coloration showing a semiquantitative amount of immunocomplexes. The cut-off control in the form of a strip that was developed with the supplied cut-off concentrate was compared to the signals produced by the serum samples. Control AG lines on the cut-off strip will be abbreviated as COC. Color reactions were categorized as “−“, less than the COC; “+”, identical to the COC; “++”, stronger than the COC; or “+++”, considerably stronger than the COC. However, the results “+”, “++”, and “+++” were all considered positive and did not make a difference in the overall result for a single sample (Table 4). COC strips were prepared according to the manufacturer’s instructions, which equaled the production of the LIA strips for serum samples. Instead of adding 15 μL of serum, 100 μL of cut-off concentrate was added to the incubation wells of the COC. For canine and equine sera, an individual cut-off strip with a cut-off concentrate specific for canine and equine samples was produced.

If the two LIAs disagreed in their results for a serum sample, both tests were performed again, and samples were evaluated anew to exclude the possibility that there was a technical error. In the following, the first round of LIAs conducted will be called the “first test series”, and the repetition of disagreeing LIAs will be named the “second test series”.

### 2.3. Statistical Methods

The intensity for each single signal on the LIA strips and the overall results were listed, organized, and analyzed for each sample with Microsoft Excel 2019 (Microsoft Corporation, Redmond, WA, USA). Color reactions of the protein-antibody-complexes for each AG line and overall results were compared for each sample. The statistical analysis was performed using the program R (R i4.1.3., R Foundation for Special Computing, Vienna, Austria). Samples were analyzed using the Cohen’s squared kappa test to give more weight to dissimilar results [38]. *κ^2^* depicts the agreement of the coloration of the single AG lines between the two LIAs for each subgroup of sera. The degree of agreement in coloration was categorized in “none to slight” (*κ^2^* < 0.2), “fair” (*κ^2^* = 0.21–0.40), “moderate” (*κ^2^* = 0.41–0.60), “substantial” (*κ^2^* = 0.61–0.80), and “almost perfect” (*κ^2^* = 0.81–1.00). Therefore, a high value for *κ^2^* can occur in negative samples, as *κ^2^* does not reflect the intensity of coloration of single AG lines but only its agreement in coloration comparing the two tests.

## 3. Results

### 3.1. Canine Serum Samples

In the first test series with both LIAs that included 200 canine sera, the agreement of the results between the two assays was 92.5% (*n* = 185). The remaining serum samples with divergent results (*n* = 13; Table 5) and serum samples for which LIA A could not automatically assign a result category due to a COC above the maximum level (*n* = 2) were then retested under the same conditions.

The accordance of the results between the two LIAs including the second test series was 94.0% in total (*n* = 188; Table 6). In the second test series, two samples tested with LIA A (i.e., RKS-B-5512-C and S98-5/1) and one sample tested with LIA B (i.e., RKS-B-5365-C) displayed different results when compared to the first test series. The automatic scan of LIA A was not successful for two strips (i.e., RKS-B-8492-C and RKS-B-5707-C) in both test series, as its COC was above the maximum level the scanner was able to handle. Those strips had to be evaluated visually by the examiner as advised in the manufacturer’s instructions. Results from those two strips agree with the results from LIA B.

**Figure 1:**
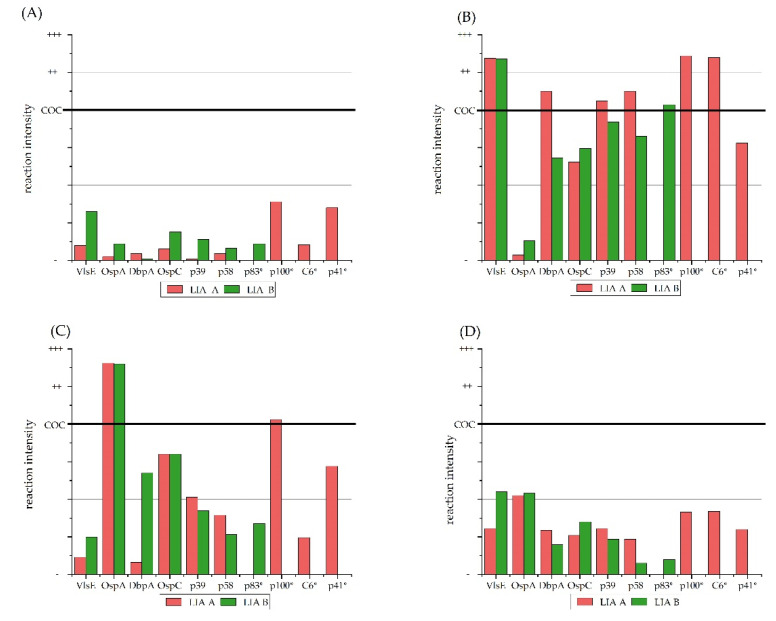
Degree of antigen-antibody reactions on LIA A and LIA B resulting from testing with canine serum samples. Ranking from reaction as “−“, less than the cut-off control COC; “+”, equal to the COC; “++”, strong; and “+++”, very strong reactions. Antigens marked with * are unique for the respective LIA. Degree of antigen-antibody reactions of sera (**A**) from group “negative”; (**B**) from group “positive”; (**C**) from group “vaccinated”; (**D**) from group “control sera”. COC, cut-off control; VlsE, variable major protein-like sequence expressed; OspA, outer surface Protein A; DbpA, decorin binding protein A; OspC, outer surface protein C; BmpA, borrelia membrane protein A; p, protein; C_6_, C_6_ peptide.

Overall, LIA A reacted stronger than LIA B with sera from the group “positive” (Figure 1B). Reactions on LIA B were stronger in color when tested with sera that originated from uninfected and vaccinated dogs (Figure 1A). The sera from the group “control sera” reacted in the same way with the antigens of the two LIAs (Figure 1D). As shown in Figure 1C, the dogs vaccinated against *Bb*sl reacted strongly to the OspA antigen of both tests.

The statistics showed significant differences in the reactions of canine serum antibodies to antigens on the two LIAs (Table 7). Results observed by the two LIAs displayed an almost perfect overall agreement of *κ*^2^ = 0.936. Regarding the four canine groups used in this study, the two LIAs agreed least in the group “negative” (*κ*^2^ = 0.219) and matched best in the group “control sera” (*κ*^2^ = 0.895). Considering AG lines in the four canine groups, the lowest agreement (*κ*^2^ = 0.084) was observed at the DbpA/p18 AG line with sera of dogs vaccinated against *Bb*sl. The OspA antigen reaction for the group “control sera” achieved the highest *κ*^2^ with a value of 0.956.

### 3.2. Equine Serum Samples

After screening 200 equine serum samples with both LIAs in the first test series, the accordance of the results was 57.5% (*n* = 115). Of these 200 equine serum samples, 17 samples (8.5%) could not be evaluated in the scanning process of LIA A, as the COC was above its maximum level. These LIA strips were then evaluated visually by the examiner as suggested in the manufacturer’s instructions. Most results disagreeing between the two LIAs were observed in the group “equivocal” (*n* = 33), followed by the group “control sera—vaccinated” (*n* = 21), the group “positive” (*n* = 20), and the group “negative” (*n* = 11). Four equine samples were recognized by the scanner as canine samples, displaying false results for RKS-B-6841-E, RKS-B-5283-E, and RKS-B-6238-E. While LIA B specifically states that OspA is a non-specific AG line in equine immunoreactions, LIA A seems to count the OspA AG line as specific for equine infections displaying equivocal or positive results for 18 horses and disagreeing results for 8 horses (Table 8). As neither the manual for LIA A nor for LIA B instructs on how to evaluate vaccinated horses, we reevaluated all horses’ sera displaying reactions for the OspA AG according to the manufacturer’s instructions for vaccinated and vaccinated and infected dogs.

Even though in the manual for LIA B a signal for OspA AG is considered non-specific for an infection with *Bb*sl-complex species, the evaluation of equine sera displaying a reaction for OspA AG resulted in many false-positive outcomes (Table 9). Nevertheless, reactions to four or more further AG lines (i.e., OspC, DbpA, BmpA, p39, or p83) occurred, which allowed the categorization of the samples as “pos” according to the manufacturer’s instruction. As the use of a lysate vaccine against LB in horses might lead to the development of antibodies against various borrelial outer surface proteins that are expressed by borreliae in vitro, immunocomplex reactions on these AG lines in combination with a reaction to the OspA AG line can occur in vaccinated animals and should—as in the evaluation scheme of dogs—not be counted as specific for infection. Only the VlsE and C_6_ AG lines can be evaluated as specific for an infection with *Bb*sl species, as VlsE is only expressed by active borreliae in vivo. Similar to dogs, horses with concurrent reactions for OspA and VlsE or C_6_ AG should be categorized as “vac + pos”.

When a reaction for OspA AG occurred on LIA strips with equine serum samples, both technical manuals supplied no information on how to evaluate these strips at the time of testing. As we were aware of the vaccination against LB, the reaction of OspA must have occurred due to the vaccination with the specific vaccine. Therefore, we categorized the equine samples according to the manufacturer’s instructions provided for dogs (Supplementary Material Table S1). This way, an accordance of 78% was reached in the group “control sera—vaccinated”.

Afterward, LIA strips exposed to equine sera and then recognized as canine samples were correctly evaluated according to the equine evaluation scheme, and samples for which OspA was considered non-specific for infection were categorized according to the evaluation scheme for dogs. In consequence, the agreement between the two LIA rose from 57.5% (*n* = 115) to 63.0% (*n* = 126).

All serum samples with divergent results (*n* = 85) were retested in a second test series under the same conditions. Thus, the comparability of the results between the two LIAs grew to 65.5% (*n* = 131); vaccinated horses were evaluated like vaccinated dogs, and LIA strips exposed to equine sera recognized as canine samples were evaluated according to the equine evaluation scheme. In the second test series, changes in three additional samples in LIA A and two additional samples in LIA B led to concurrent results. Additionally, the equine samples in which the COC was in the first test run too high for evaluation could now be evaluated with the scanner aside from two horses that displayed a COC too high for evaluation in both test series (S4-28 and RKS-B-10899-E). These horses were then evaluated visually by the examiner. As already described in the first test series, a few horses’ sera were still recognized as dog samples, of which three sera were evaluated as canine in both test series (RKS-B-6841-E, RKS-B-5283-E, and RKS-B-6238-E).

The group “equivocal” (*n* = 50) displayed the most discrepancies (58%), followed by the groups “positive” (36%), “negative” (20%), and “control sera—vaccinated” (20%).

**Figure 2:**
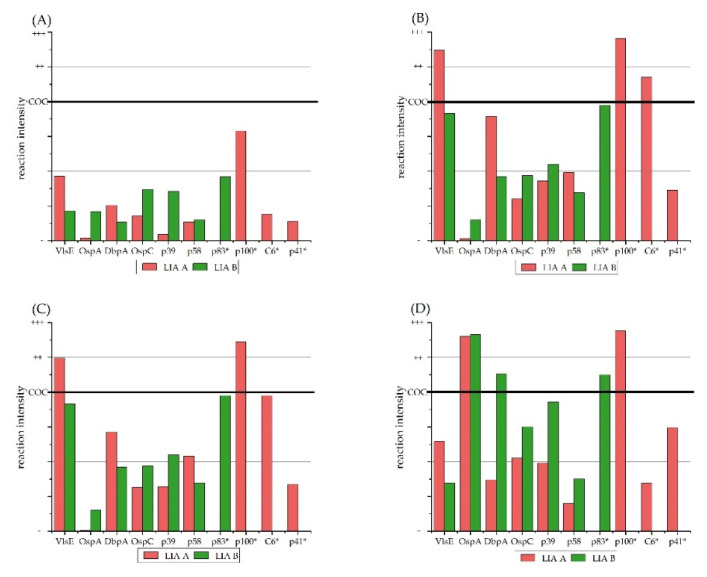
Degree of antigen-antibody reactions on LIA A and LIA B resulting from tests with equine serum samples. Ranking from reaction as “−”, less than the cut-off control COC; “+”, equal to the COC; “++”, strong; and “+++”, very strong reactions. Antigens marked with * are unique for the respective LIA. Degree of antigen-antibody reactions of sera (**A**) from group “negative”; (**B**) from group “positive”; (**C**) from group “equivocal”; (**D**), from “control sera—vaccinated”. COC, cut-off control; VlsE, variable major protein-like sequence expressed; OspA, outer surface Protein A; DbpA, decorin binding protein A; OspC, outer surface protein C; BmpA, borrelia membrane protein A; p, protein; C_6_, C_6_ peptide.

Considering the extent of coloration of AG lines on the LIA strips, the p100-antigen line on LIA A developed the strongest reaction compared to all other antigens of both LIAs in all four groups (Figure 2); further, p100 was equal to or higher than the COC on 170 of the 200 equine LIA strips. LIA A produced a slightly stronger color reaction in the group “negative” (Figure 2A). Both LIAs showed comparable antibody reactions for groups “positive” and “equivocal” (Figure 2B,C); serum samples from group “equivocal” showed stronger signals for the OspC and p39 AG on LIA B, while p58 and DbpA were stronger on LIA A. As illustrated in Figure 2D, samples from “control sera—vaccinated” showed strong signals for OspA on both LIAs.

The statistical analysis (Table 7) shows significant differences in the antibodies’ reaction patterns for single serum samples based on the results derived from the two LIAs. Compared to canine sera, the rate of concordance for equine serum samples was generally lower. All equine results displayed a substantial comparability at *κ*^2^ = 0.808. Results for the group “control sera—vaccinated” displayed the highest degree of comparability (*κ*^2^ = 0.519), except for the DbpA/p18 signal (*κ*^2^ = 0.015). The results for the group “equivocal” agreed the least (*κ*^2^ = 0.223), considering the signals reactions to DbpA/p18 (*κ*^2^ = 0.186) and OspA (*κ*^2^ = 0.083) were the least comparable. Groups “positive” and “negative” showed a moderate degree of comparability with *κ*^2^ = 0.450 and *κ*^2^ = 0.457, respectively.

## 4. Discussion

This study was designed to compare two LIAs regarding sensitivity, specificity, overall results, and laboratory handling of canine and equine serum samples.

### 4.1. Sensitivity, Specificity, and Overall Results

For LB in dogs, it is essential to correctly detect silent carriers of borreliae, as dogs and owners live in a close relationship [39]. The estimated growth of the tick population due to more favorable climate conditions in the next years will lead to a higher number of canine infections [40]. When dogs are neither vaccinated nor protected or tested and treated against LB, tick attachment and the possible infection of uninfected nymphal stages on these infected dogs will allow more infected ticks to reach gardens and greens, where deer and other wildlife are not residing. Nymphs will then molt to adults and possibly bite and may infect humans. On the other hand, a false-positive diagnosis of LB will lead to the unnecessary treatment of dogs with antibiotics with unnecessary side effects and the risk of antibiotic-resistance development in bystander bacteria.

In this context, both LIAs evaluated in his study seem to be highly comparable (94.0%) and appropriate for diagnostic purposes with canine serum samples. For the group of “canine control sera”, the best agreeance between the results of the two LIAs could be observed (*κ^2^* = 0.895). Both LIAs produced twelve serum samples from the group “canine control sera—positive” identified correctly as “pos” and also nineteen serum samples from the group “canine control sera—negative” accurately identified as “neg”. Both LIAs allocated one serum sample each from the “canine control sera—vaccinated” as falsely “neg” and the residual nineteen serum samples correctly as “vac” (Table 6). As the “canine control sera” originate from dogs with a confirmed infection status, and both LIA tested different vaccinated canine serum samples as “neg”, we could clearly identify these two results as false. The group “positive” (*n* = 50) displayed the second-best agreeance (*κ^2^* = 0.882). However, here, LIA A tested 47 serum samples correctly as “pos”, while LIA B tested 45 serum samples correctly as “pos” (Table 6). This disagreement between the previous allocation and the LIAs might be due to the previously used tests or might even be a previous case of human error. Next, the second-least agreeance of the results between the two LIAs (*κ^2^* = 0.489) was observed for the group “vaccinated” (*n* = 50) even though both LIAs allocated the 50 serum samples correctly as “vac” (Table 6). The results for the group “negative” (*n* = 50) agreed with *κ^2^* = 0.219, while LIA A allocated 47 serum samples correctly as “neg” and LIA B 48 serum samples as “neg”; both produced two “false-positive” in this group. Of these “pos”-labeled samples, one was “pos” in both LIAs with 256.6 KELA units (Table 6). The other two “pos” dogs had either 138.9 or 151.7 KELA units. In this case, it is difficult to conclude which test produced an accurate result. In general, seronegative dogs display KELA levels below 100 KELA units. Thus, it is reasonable to conclude that sample with agreeing “pos” results from the two LIAs might have been allocated falsely beforehand. Further, we observed that on LIA A, canine serum samples reacted less strongly with VlsE than on LIA B in groups “negative”, “vaccinated”, and “control sera”. However, in the previous three groups, a stronger reaction against the C_6_ peptide was visible. In the group “positive”, both LIAs produced strong color reactions to VlsE and in LIA A equally strong color reactions to C_6_. Therefore, in our opinion, separating C_6_ and VlsE does not add extra value to the performance of a LIA, which disagrees with a study conducted with dogs by Breu and Müller (2017) [41]. In this study, over 25% of 236 positive canine sera displayed disagreeing results between the C_6_ and the VlsE AG [41]. In our study, only 8 of the 200 canine serums samples (4%; RKS-B-7240-C, RKS-B-8484-C, RKS-B-5813-C, RKS-B-7973-C, RKS-B-7996-C, RKS-B-8324-C, S98-5/1, and S98-5/4) showed diverging results between the C_6_ and the VlsE AG on LIA A. On LIA B, three of these five dog sera (RKS-B-8324-C, RKS-B-7669-C, and S98-5/1) produced different results when compared to LIA A (Table 6). However, in our opinion, both tests are highly suitable for LB diagnostic for dogs, and the addition of the C_6_ antigen does not influence the results negatively.

In horses, the existence of clinical LB is controversially discussed, and if it exists, clinical signs are highly diverse, ranging from neuroglial disorders to lameness, uveitis, and cutaneous pseudolymphoma [42,43,44]. Yet, many studies in various countries around the world describe and confirm infections of horses with *Bb*sl organisms [45,46,47]. Like dogs, horses are a part in the infectious chain of *Bb*sl-complex species [48]. However, equines probably play a lesser role in the indirect transmission to humans, as horses and humans live in a remoter relationship than humans and dogs. Due to the recommendation to treat only seropositive horses with antibiotics that at the same time display clinical signs and in which all other diseases can be ruled out [42], the impact of false-negative results is not as high as in dogs.

For equine serum samples, the two LIAs displayed concordant results for 65.5% of the sera. Again, the results from the group “control sera—vaccinated” agreed the most (*κ^2^* = 0.519), with 47 serum samples correctly allocated as “vac” by LIA A and 48 serum samples correctly allocated as “vac” by LIA B. The second-best agreeance was observed for the group “negative” (*κ^2^* = 0.457). Here, LIA B displayed 47 “neg” results, while LIA B produced only 38 “neg” (Table 10). The 50 samples in group “positive” had been assigned to this group by evaluating these sera with the assay “Borrelia Veterinär plus OspA LINE” (Virotech GmbH) or its predecessor model. In our study, 17 of these sera tested positive on LIA B and 29 on LIA A (Table 10). An explanation for the discrepant test results could be the use of a predecessor model of the “Borrelia Veterinär plus OspA LINE” (Virotech GmbH), a Western blot used between 2006 and 2011. This WB contained 16 AG lines, which is 9 AG lines more than current LIA B. Reactions with those nine antigens might have contributed to more positive results. For some of these proteins, e.g., the p41, cross-reactions with spirochetes such as relapsing fever borreliae have been described, which limits their diagnostic value [30,49,50]. Another explanation for discrepant results of sera could be the subjective assessment by the technical person in the case of LIA B and the predecessor model of Borrelia Veterinär plus OspA LINE (Virotech GmbH), making human error more likely in these tests. LIA A’s evaluation via a scanning system may prevent varying results, particularly if more than one person evaluates the assays. In the group “equivocal”, the highest number of diverging results (*n* = 29) occurred, and the color reaction and overall results were the least comparable between the two LIAs (Table 7). LIA A produced 25 seropositive hoses, while LIA B produced only 6. Considering all samples, LIA A produced a total of 77 positive serum samples, while LIA B produced a total of 32.

High numbers of false-positive samples are concerning and show that the serologic assessment of equine sera for *Bb*sl-specific antibodies is challenging and should be improved to reduce the overdiagnosis and unrequired antibiotic treatment of horses. This is especially important for the animal, as many long-term antibiotic treatment regimens can lead to the development of diarrhea, colitis, and might even cause the needless death of the equine [51,52]. Further, long-term antibiotic treatment in horses is generally performed by intravenous or intramuscular injection of the compound, as almost all antibiotics recommended and available for equine LB [42]—except for tetracycline—must be applied parenterally. Continuous intravenous or intramuscular injection often leads to thrombophlebitis [53] and may induce abscesses.

The consideration of OspA AG as specific for borrelial infection is probably the main disadvantage of LIA A [17,54]. The development of antibodies against OspA is specific for vaccination [32], as borreliae express OspA only while residing in the tick’s intestine [55]. When *Ixodes* spp. ticks start to feed blood on the mammalian host, borreliae change their outer-surface antigen expression pattern, especially from OspA to OspC [56]. Thus, the host will encounter not many *Bb*sl organisms expressing OspA during the transmission and thus will not develop antibodies against OspA due to the infection [34]. In contrast, for canine serum samples both technical manuals provide precise evaluation schemes. Dogs with OspA-specific antibodies and against antigens other than VlsE/C_6_ are then considered “vac”, and when the VlsE/C_6_ AG is also visible, the sera come from “vac + pos” dogs. Since lysate vaccines against LB were used in the horses [37] that were included in this study, antibody reactions to various borrelial outer surface proteins must be considered [35]. In contrast, antibodies against VlsE and C_6_ antigens are associated with an active infection with *Bb*sl organisms, as VlsE is expressed in vivo only [27]. Further, the *vls* gene is expressed on the linear plasmid lp28-1 [57], which might get lost during passaging of *Borrelia* spp. in vaccine production [58]. When this plasmid is lost, borreliae will therefore lose the ability to express the VlsE lipoprotein, and thus, no antigen reaction to VlsE occurs in vaccinated animals [37,59,60]. This assumption would allow the evaluation of vaccinated horses according to the canine evaluation protocol; however, further studies with a larger number of experimentally vaccinated horses might be helpful.

We furthermore recommend reducing the number of AG lines, including only these that are highly specific for infection or vaccination with *Bb*sl.

**Figure 3:**
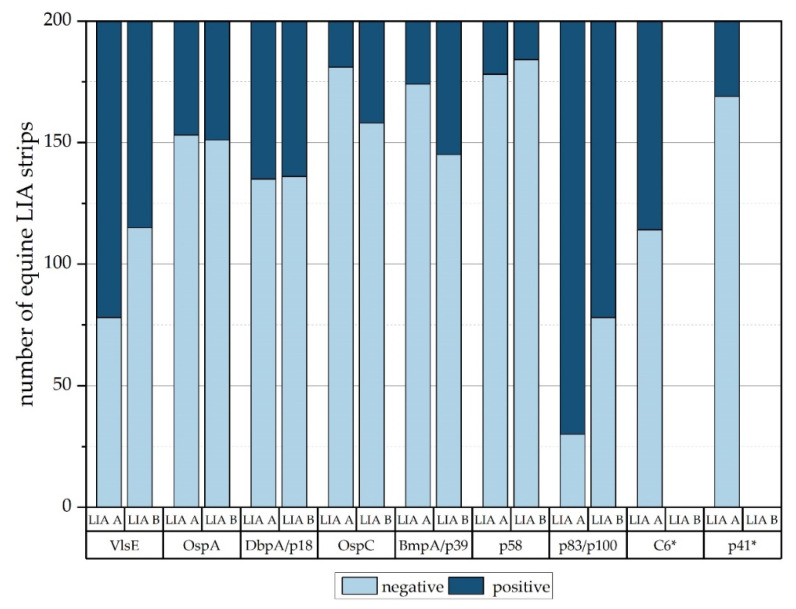
Number of equine AG lines that displayed positive (equal or higher than COC) or negative color reactions on the LIA strips of both LIAs. Antigens marked with * are unique for the respective LIA.

p100 and p83 are associated with either the protoplasmic cylinder [61] or the flagella [62] of borreliae and are highly sensitive antigens for late stages of *Bb*sl infection [20,63]. p83/100 are similar for *Bb*ss and *B. afzelii* although these proteins show differences when compared to *B. garinii* [64]. However, in a comparison of the N-terminal amino acid sequences of p100 and p83, no differences in the amino acid sequence were found, and it was concluded that p100 and p83 are identical [65]. In this study, p100 used on LIA A showed the strongest color reactivity in all equine serum test groups. LIA A strips incubated with equine sera produced reactions to p100 in 170 cases, which accounts for 85.0% of the 200 horses. This non-specificity of p100 might be one reason for a high portion of horses identified as “pos” (40.7%) compared to LIA B with only 23.3%. However, when we compared the signals of the similar p83 AG on LIA B, a disproportionate high number of reactions to this AG (*n* = 122; 61%) was observed as well. Consequently, we compared the numbers of reactions to the p100 and p83 AG line with the those to other AG lines found on the respective LIA (Supplementary Material Table S2 and Figure 3).

When all color reactions on the AG lines (i.e., degree of color reaction (Figure 1 and Figure 2) and overall number of reactions (Figure 3)) are considered, it seems that the p83 and p100 AG react very often and may falsely call for a *Bb*sl infection in horses and thus should be omitted from diagnostic LIA strips to avoid false-positive results in horses.

Of all 200 tested equine samples, only two samples (RKS-B-5442-E, RKS-B-5558-E) reacted as positive for the C_6_ and negative for VlsE on LIA A. One of the two samples (RKS-B-5442-E) was diagnosed “negative” in LIA B but equivocal in LIA A, while the other sample (RKS-B-5558-E) was categorized as “equivocal” in both LIAs. On the other hand, the VlsE AG appeared isolated without a reaction against C_6_ in 37 equine serum samples. To the best of our knowledge, there is no literature available describing the heterogenicity of equine immune responses to VlsE or C_6_. The C_6_ peptide is a synthetically produced part (IR6) of the VlsE lipoprotein. Regarding different invariable regions of the VlsE lipoprotein, X-ray crystallography of VlsE displayed a limited surface exposure of IR6 [66]. This study suggests that in horses, more antibodies bind to the complete VlsE surface than to the limited surface of C_6_ peptide. Therefore, in the authors’ opinion, there seems to be no additional value and probably no considerable disadvantage of including C_6_. However, as depicted in Figure 3, considerably more LIA A strips displayed a reaction to VlsE than LIA B strips (LIA A: *n* = 122; LIA B: *n* = 85). This observation might indicate that the reactivity to the VlsE AG is too high in LIA A, as only 50 horses were seropositive, and 50 additional horses were equivocal. This might be an additional reason why LIA A produces more positive results than LIA B (Table 10). In conclusion, the interpretations of results from LIAs exposed to equine sera are still challenging, and improvements concerning OspA and the selection of AG are needed.

### 4.2. Handling of LIAs

In terms of handling and implementation of the tests in the laboratory, there are hardly any differences. LIA A features a longer incubation time compared to LIA B (45 min for serum and conjugate versus 30 min); the incubation time for the substrate is two minutes shorter for LIA A (ten minutes versus twelve minutes). An advantage of LIA A is its multi-species conjugate; in contrast to LIA B, the test kit from LIA A contains one conjugate used for both canine and equine serum samples. Furthermore, it is unnecessary to mix the conjugate from LIA A with washing buffer and conjugate concentrate before testing. In our view, the workflow of LIA A is more user-friendly.

LIA A provides a scanner for the evaluation scheme. During the scanning process, a combined evaluation sheet of all samples and an extra folder for each sample and each strip with its AG lines’ color intensity are created. After scanning, the color reaction for each AG line is displayed as a number from 0 to 9. In contrast, LIA B is evaluated visually by the examiner by comparing the AG line on the test strip with the COC. In the authors’ opinion, the evaluation via a scanning system is the main advantage of LIA A, allowing a precise evaluation of samples and thus comparability. LIA B is prone to human error, and evaluation might change according to the evaluator and the light conditions. Therefore, the evaluation as conducted in LIA A seems to be most suitable for the scientific field, as human error is mostly ruled out, and the number values provided by the program allow a more precise and easy statistical evaluation. A further advantage of LIA A is the fact that results for each sample are displayed in an extra folder, and this sheet can be sent directly to the veterinary clinic or diagnostic facility as a medical report. Then again, when the scanning process for LIA A fails—as it did in this study in 2 dogs and 17 horses—the visual evaluation by the examiner is challenging, as the COC is on the same strip as the sample AG lines. A visual examination and thorough comparison were possible only for the first two to three AG lines next to the COC; all other AG lines could only be judged imprecisely. Furthermore, LIA A’s scanning system had problems when strips were incubated with hemolytic sera. These hemolytic sera produced speckles on the nitrocellulose membrane concealing the protein signals. In hemolytic sera, the concentration of intracellular components of erythrocytes and other blood cells is released into the extracellular space of the blood [67]. These components may bind on the nitrocellulose membrane of a LIA strip [68]. The nitrocellulose membrane of LIA A seemed to be affected more strongly by this phenomenon than the membrane of LIA B. These speckled discolorations complicated the identification of antigen-antibody reactions in LIA A. In the scanning process of these speckled strips, equine samples were identified as canine samples as the canine control band seemingly reacted, or the scanner mistook a speckle as an antigen-antibody-complex reaction.

Dried strips of LIA B showed a slight purple discoloration. However, this had no impact on the evaluation.

## 5. Conclusions

Both tests are reliable assays for the diagnosis of LB in dogs, considering an agreement of 94.0%. For equine serum samples, the agreement was 65.5%. This result clearly shows that the serologic diagnosis of LB with equine sera is still challenging. Both LIAs should improve their interpretation of sera from vaccinated horses, and especially in the case of LIA A, the consideration of the OspA AG as specific for infection must be changed (current information on the LIA A manufacturer’s website reconsiders the OspA AG role during *Bb*sl infection, and an evaluation scheme for vaccinated horses has been provided). Regarding test specificity, LIA B seems to be more reliable and produced fewer false-positive results. The test protocols are similar and allow no preference for a specific LIA. The evaluation of strips by a scanner makes LIA A the more adequate choice in a scientific background, as it is not prone to human error and displays higher comparability of evaluations and easier application for statistics. However, the evaluation scheme for LIA A in the case of vaccinated horses still needs improvement.

## Figures and Tables

**Table 1:** Serostatus of canines and equines used in this study.

	Seropositive	Equivocal	Seronegative	Vaccinated
**Canine test sera** **(*n* = 150)**	50		50	50
Canine control sera—positive/vaccination/negative (*n* = 50) *	12		19	19
**Equine test sera** **(*n* = 150)**	50	50	50	
**Equine control sera—vaccination** (*n* = 50) **				50

* Dogs used for studies with *Bb*sl infections at Cornell University (Ithaca, NY, USA) [34,35,36]. ** Horses experimentally vaccinated with the vaccine EquiLyme^®^ (Boehringer Ingelheim Vetmedica GmbH, Ingelheim am Rhein, Germany) [37].

**Table 2:** Recombinant antigens sprayed on the nitrocellulose strips of both LIAs.

Antigen	LIA A	LIA B
VlsE	*Ba*	*Bb*ss, *Bg*
OspA	*Ba*	*Ba*, *Bg*, *Bb*ss
DbpA/p18	*Bb*ss, *Bg*, *Ba*, *Bs*	*Bg*, *Bbav*, *Ba*
OspC/p23	*Ba*, *Bb*ss, *Bs*, *Bg*, *Bg*II	*Ba*, *Bbav*, *Bb*ss
BmpA/p39	*Ba*	*Ba*
p58	*Bg*	*Bbav*
p83	np	*Ba*
C_6_	*Bg*	np
p41	*Bb*ss	np
p100	*Ba*	np

Antigens derived from the following genospecies: *B. afzelii* (*Ba*), *B. burgdorferi* sensu stricto (*Bb*ss), *B. bavariensis* (*Bbav*), *B. garinii* (*Bg*), *B. garinii II* (*BgII*; probably *B. bavariensis*), *B. spielmanii* (*Bs*), not present on the assay (np). VlsE, variable major protein-like sequence expressed; Osp, outer surface protein; DbpA, decorin binding protein A; BmpA, borrelia membrane protein A; p, protein.

**Table 3:** Allocation of serum samples based on results obtained with LIA A and to its manufacturer’s instructions.

Allocation Categories	Canine Sera	Equine Sera
**neg**	0–1 AG lines ≥ COC except OspA or VlsE/C_6_ AG line	0–2 AG lines ≥ COC except OspA or VlsE/C_6_ AG line
**equiv**	2–3 AG lines ≥ COC except OspA or VlsE/C_6_ AG line	VlsE/C_6_ AG line and 0–2 AG lines ≥ COC or 3 AG lines except VlsE AG line ≥ COC
**pos**	VlsE/C_6_ AG line or ≥ 4 AG lines (except OspA AG line) ≥ COC	VlsE/C_6_ AG line and ≥ 3 AG lines ≥ COC or ≥ 4 AG lines ≥ COC or VlsE/C_6_ AG line and p18 AG line and ≥ 1 AG line ≥ COC
**vac**	OspA AG line and ≥ 0 AG lines (except VlsE/C_6_) ≥ COC	-
**vac + pos**	OspA AG line and VlsE/ C_6_ AG line and ≥0 AG lines ≥ COC	-

VlsE and C_6_ are considered as one AG line; however, the appearance of one of the two lines is enough to allocate samples to the categories “equiv” and “pos” as well as “vac + pos”. Horses with an OspA AG line were evaluated once according to the evaluation protocol for samples from horses with the antigen-antibody reaction on the OspA AG line and once again according to the evaluation protocol for vaccinated and vaccinated and positive dogs. AG line, antigen line; COC, cutoff control AG line.

**Table 4:** Allocation of serum samples based on results obtained with LIA B and to its to the manufacturer’s instructions.

Allocation Categories	Canine Serum	Equine Serum
**neg**	0 AG lines or AG lines ≤ COC or VlsE AG line = COC or 0–1 AG line (except VlsE and OspA) ≥ COC	0 AG lines or AG lines ≤ COC or 0–2 AG line (except VlsE) ≥ COC
**equiv**	2–3 AG lines ≥ COC (except VlsE and OspA)	3 AG line (except VlsE) ≥ COC or VlsE AG line and 0–2 AG lines ≥ COC
**pos**	VlsE > COC or VlsE ≥ COC and 1 AG line (except OspA) or ≥ 4 AG lines (except VlsE and OspA)	VlsE AG line and ≥ 3 AG lines ≥ COC or VlsE AG line and DbpA AG line and 1 AG line or ≥ 4 AG lines ≥ COC (except VlsE)
**vac**	OspA AG line isolated or OspA AG line and ≥ 1 AG line (beside VlsE) or OspA AG line and VlsE AG line isolated = COC	-
**vac + pos**	OspA AG line and VlsE AG line isolated ≥ COC or OspA AG line and VlsE AG line and ≥ 1 AG line	-

OspA AG line was considered as non-specific AG line in equine immunoreactions. Equine serum samples with an OspA AG line were evaluated once according to the equine evaluation protocol by simply considering the OspA AG line as non-specific for equine immunoreactions and thus not counting this AG line and afterward evaluated according to the evaluation protocol for vaccinated or vaccinated and infected dogs. AG line, antigen line; COC, cutoff control AG line.

**Table 5:** Canine samples with divergent results in the two LIAs. KELA-results below 100 are considered as negative. KELA levels above 100 need confirmation by a LIA.

Sample ID	Group	LIA A	LIA B	KELA Values
RKS-B-5365-C	Negative	neg	pos	138.9
RKS-B-7240-C	Negative	pos	neg	151.7
RKS-B-7279-C	Negative	vac	neg	125.6
RKS-B-8564-C	Negative	neg	pos	127.0
RKS-B-5119-C	Positive	pos	vac + pos	394.6
RKS-B-7996-C	Positive	pos	equiv	103.7
RKS-B-8113-C	Positive	pos	equiv	243.1
Hanka	Vaccinated	vac + pos	vac	613.6
RKS-B-5512-C	Vaccinated	neg	vac	234.9
RKS-B-8324-C	Vaccinated	vac + pos	vac	580.9
S98-5/1 *	Control sera	pos	neg	63.3
A93-3/3 *	Control sera	neg	vac	383.2
A93-3/4 *	Control sera	vac	neg	311.2

* Dogs used for *Bb*sl infection studies at Cornell University (Ithaca, NY, USA) [34,35,36].

**Table 6:** Defined *Bb*sl-serostatus of dogs (Groups; *n* = 200) and allocated results after the second round of serologic testing using LIA A and LIA B.

	Groups	Positive (*n* = 50)	Negative (*n* = 50)	Vaccinated (*n* = 50)	Control Sera (*n* = 50) ^1^
LIA Results					
**LIA A**	**pos**	**47**	2	0	12
	**equiv**	0	0	0	0
	**neg**	3	**47**	0	20
	**vac**	0	1	50 **	18
**LIA B**	**pos**	45 *	2	0	12
	**equiv**	2	0	0	0
	**neg**	3	**48**	0	20
	**vac**	0	0	50 ***	18

* One infected dog showed additional AG lines for vaccination. ** Three vaccinated dogs showed additional AG lines for infection. *** One vaccinated dog showed additional AG lines for infection. ^1^ Dogs used for *Bb*sl infection studies at Cornell University (Ithaca, NY, USA) [34,35,36].

**Table 7:** *κ^2^* for comparable antigen-antibody-complex signals on LIA A and B when canine or equine sera were applied. The degrees of agreement are categorized into “none to slight” (*κ^2^* < 0.2), “fair” (*κ^2^* = 0.21–0.40), “moderate” (*κ^2^* = 0.41–0.60), “substantial” (*κ^2^* = 0.61–0.80), and “almost perfect” (*κ^2^* = 0.81–1.00). A high value for *κ*^*2*^ does not represent a high reaction intensity but merely a high agreement between the two LIAs.

Antigen Group	VlsE	OspA	DbpA/p18	OspC	BmpA/p39	p58
**can_neg_**	0.236	0.111	0.331	0.123	0.560	**0.580**
**can_pos_**	**0.852**	0.147	0.156	0.645	0.850	0.574
**can_vacc_**	0.411	0.460	−0.084	**0.772**	0.577	0.464
**can_con_**	0.777	**0.956**	0.096	0.805	0.785	0.508
**equ_neg_**	**0.575**	0.123	0.572	0.254	0.169	0.414
**equ_pos_**	**0.636**	0.084	0.358	0.315	0.565	0.445
**equ_vacc_**	0.665	**0.668**	0.015	0.559	0.523	0.234
**equ_equiv_**	0.367	0.083	0.186	**0.560**	0.551	0.459

**can_neg_**, canine sera from the group “negative”; **can_pos_**, canine sera from the group “positive”; **can_vacc_**, canine sera from the group “vaccinated”; **can_con_**, canine sera from the group “control sera” [34,35,36]; **equ_neg_**, equine sera from the group “negative”; **equ_pos_**, equine sera from the group “positive”; **equ_vacc_**, equine sera from the group “control sera—vaccinated”; **equ_equiv_**, equine sera from the group “equivocal”; VlsE, variable major protein-like sequence expressed; OspA, outer surface protein A; DbpA, decorin binding protein A; OspC, outer surface protein C; BmpA, borrelia membrane protein A; p, protein. The highest values for κ2 for each AG line and subgroup are written in bold.

**Table 8:** Equine serum samples with divergent results in the LIA A due to the recognition of the OspA band as specific for *Bb*sl-infection.

Sample ID	Group	LIA A with OspA *	LIA A without OspA **	LIA B	KELA Values
S4-8 ^1^	Control sera—vaccinated	equiv	neg	neg	654.3
S4-32 ^1^	Control sera—vaccinated	pos	equiv	equiv	735.0
S4-180 ^1^	Control sera—vaccinated	pos	equiv	equiv	688.6
S7-43 ^1^	Control sera—vaccinated	equiv	neg	neg	728.9
S7-44 ^1^	Control sera—vaccinated	pos	equiv	neg	633.8
S7-58 ^1^	Control sera—vaccinated	equiv	neg	neg	725.4
S7-61 ^1^	Control sera—vaccinated	pos	equiv	neg	694.8
S1-5 ^1^	Control sera—vaccinated	equiv	neg	pos	568.4

* Evaluation of LIA A strips according to the manufacture’s instruction considering OspA AG lines indicative for infection, ** Evaluation of LIA A strips according to the manufacture’s instruction considering OspA AG lines not indicative for infection and not for vaccination. ^1^ Horses experimentally vaccinated with the vaccine EquiLyme^®^ (Boehringer Ingelheim Vetmedica GmbH, Ingelheim am Rhein, Germany) [37].

**Table 9:** Equine serum samples with OspA AG signals and results considered “pos” on LIA B due to the recognition of ≥ 4 AG lines (beside VlsE and OspA) that are considered as non-specific for an infection with *Bb*sl complex species in vaccinated dogs.

Sample ID	Group	LIA A	LIA B	KELA Values
S4-5 ^1^	Control sera—vaccinated	pos	pos	584.0
S4-26 ^1^	Control sera—vaccinated	pos	pos	659.9
S4-30 ^1^	Control sera—vaccinated	pos *	pos	709.4
S4-31 ^1^	Control sera—vaccinated	pos	pos	697.8
S4-176 ^1^	Control sera—vaccinated	pos **	pos	722.4
S7-86 ^1^	Control sera—vaccinated	pos *	pos	731.8
S1-5 ^1^	Control sera—vaccinated	equiv	pos	568.4
S4-28 ^1^	Control sera—vaccinated	neg	pos	661.2
S5-31 ^1^	Control sera—vaccinated	neg	pos	646.9

* LIA strips with a reaction to the VlsE or C_6_ AG; ** only case tested with LIA A; reactions to four AG lines beside VlsE and OspA were considered as “pos”. ^1^ Horses experimentally vaccinated with the vaccine EquiLyme^®^ (Boehringer Ingelheim Vetmedica GmbH, Ingelheim am Rhein, Germany) [37].

**Table 10:** Defined serostatus of horses (groups; *n* = 200) and allocated results using LIA A and LIA B. (Specific samples were tested two time as described above.)

	Groups	Positive (*n* = 50)	Equivocal (*n* = 50)	Negative (*n* = 50)	Control Sera-Vaccinated ^1^ (*n* = 50)
LIA Results					
**LIA A**	**pos**	**29**	**25**	5	0
	**equiv**	20	22	6	1
	**neg**	1	3	**38**	2
	**vac**	0	0	0	47 *
**LIA B**	**pos**	17	6	1	0
	**equiv**	**28**	**23**	2	1
	**neg**	4	21	**47**	1
	**vac**	1 *	0	0	48 **

* 18 vaccinated horses also showed signals for infection. ** 8 vaccinated horses also showed signals for infection. ^1^ Horses experimentally vaccinated with the vaccine EquiLyme^®^ (Boehringer Ingelheim Vetmedica GmbH, Ingelheim am Rhein, Germany) [37].

## Data Availability

Not applicable.

## Supplementary Materials

The following supporting information can be downloaded at: https://www.mdpi.com/article/10.3390/vetsci9110633/s1, Table S1: Equine LIA strips evaluated according to manufacturer’s instructions for canines; Table S2: Positive and negative reactions of AG lines on both LIAs.
